# Participation for mental health service development in China: Conditions, challenges, facilitators, and outcomes

**DOI:** 10.1002/ajcp.70026

**Published:** 2025-11-14

**Authors:** Zhiying Ma, Yu Fan, Xiyuan Chen, Lindsay Sheehan, Sang Qin, Aoxuan Cao, Liang Zhou

**Affiliations:** ^1^ Crown Family School of Social Work, Policy, and Practice University of Chicago Chicago Illinois USA; ^2^ The Affiliated Brain Hospital of Guangzhou Medical University Guangzhou Guangdong China; ^3^ Department of Psychology Illinois Institute of Technology Chicago Illinois USA; ^4^ Department of Rehabilitation Psychology and Special Education, School of Education University of Wisconsin ‐ Madison Madison Wisconsin USA

**Keywords:** China, participatory process, paternalism, peer support, serious mental illnesses

## Abstract

This study analyzes a participatory project to develop peer support services for people with serious mental illnesses (SMIs) in China. Drawing on interviews with psychiatrists, social workers, service users, and a family caregiver, it examines the conditions, challenges, facilitators, and outcomes of participation in a paternalistic context unfamiliar with such approaches. Participation was made possible by local calls for change, personal trust, and institutional endorsements. Challenges included service users' difficulties understanding technical materials, reticence in group settings, technology barriers, and limited institutional support. To address these, professionals adjusted meeting formats and communication styles, offered emotional and material support, and helped service users engage with project content. Participants recommended including government officials and expanding the team's diversity in future efforts. The process produced a culturally responsive manual integrating diverse stakeholder perspectives, particularly those of service users. It also fostered emerging shifts in professional reflexivity, service user empowerment, and mutual relationships, setting the stage for second‐order change. However, participation remained shaped by structural inequalities and lacked sufficient institutional backing for long‐term transformation. This case highlights the potential and limits of participatory processes in hierarchical systems and offers strategies to make them more inclusive and transformative for disabled populations in China and beyond.

## Highlights

- Studied participatory service development in China's paternalistic mental health system.
- Participation hinged on change‐makers, trust, and backing from institutional authorities.
- Challenges included service user reticence, comprehension, and tech issues rooted in marginalization.
- Professionals supported participation by offering space, respect, and individualized support.
- Participation shaped responsive services and shifted attitudes, capacities, and relationships.

## INTRODUCTION

### Development of community mental health and peer support services

This article analyzes a project of participatory development of community mental health services, specifically peer support for persons with serious mental illnesses (SMIs), in China. Peer support, well‐established in countries like the United States, Canada, the United Kingdom, and Australia, typically involves trained, often paid, individuals with lived experiences of mental illnesses providing support to others (Corrigan et al., 2008). It emerged from the mental health consumer/survivor/ex‐patient (c/s/x) movement in the 1970s, as ex‐patients protested the dehumanizing treatment in hospitals and argued that they were best equipped to address each other's needs (Davidson et al., 2006). As psychiatry becomes deinstitutionalized and community‐based, peer support has been increasingly recognized and adopted by mainstream mental healthcare (Davidson et al., 2012).

SMIs are a category of persistent psychiatric disorders, such as schizophrenia and bipolar disorder, that significantly impact behavior, cognition, and social relationships. Rather than focusing on the disease, community psychology emphasizes the stigma, marginalization, and loss of opportunity experienced by people with SMIs (Corrigan & Penn, 1999; Kloos, 2005). Peer support can empower individuals with SMIs by centering on their experiential knowledge, repositioning them as helpers and leaders, enhancing their self‐worth and sense of belonging, and facilitating the development of resources and skills (Brown, 2009). However, scholars warn of the risk of bureaucratic co‐optation when peer support is integrated into formal systems (Borkman, 1990), and advocate for participatory, culturally responsive approaches to service design (Fang et al., 2024).

There have been efforts to develop peer support in non‐Western countries (e.g., Lee et al., 2019; Pathare et al., 2018; Tse et al., 2014), but the model's emphasis on autonomy and activism may clash with local values. In China, psychiatry remains paternalistic: because service users are seen as lacking insight, psychiatrists and families often make decisions for them (Ma, 2025). Moreover, service users are often seen as dangerous, incompetent, or morally deficient, resulting in profound social exclusion and internalized shame (Xu et al., 2018). Even family caregivers and psychiatrists may hold stigmatizing views against their relatives and patients (Chen et al., 2023; Wang, Meng, et al., 2023). The deep‐seated stigma and marginalization may explain the rarity of self‐help groups among persons with SMIs, despite their presence in other disabled populations (He & Huo, 2013).

China's psychiatric services are historically institution‐based. Since 2004, the state has been actively developing community mental health services, which have been included in the National Basic Public Health Services since 2009 (Qin, 2017). They involve regular visits by community practitioners to monitor symptoms, medication, and the risk of violence. These biomedically oriented services are insufficient in combating stigma or promoting recovery (Wang, 2023). In recent years, governmental and societal efforts have supported community‐based rehabilitative services, including peer support. For example, since 2013, psychiatrists have organized peer support among individuals with SMIs in Beijing and several other cities (Fan et al., 2018). Compared to Western counterparts, these programs are usually designed and led by doctors or social workers. They often treat peer supporters as volunteers, limiting their roles to sharing recovery stories or organizing leisure activities. It is unclear how those programs were developed, but to some extent, they still reflect the professional paternalism dominant in China.

As anthropologists and transcultural psychiatrists remind us, culture is not an ahistorical, unified whole, but rather a set of relations and meanings suffused with power dynamics and subject to change (Kirmayer, 2012; Ortner, 2006). Culturally responsive services should incorporate the voices of marginalized groups and strive to enhance their well‐being in ways that are feasible and safe within the specific context. Including marginalized groups and transforming the status quo also lie at the heart of peer support. Therefore, for peer support to be culturally relevant in a new context like China, stakeholder perspectives—especially of service users—must shape service design.

### Participatory processes in health research and service development

Participatory processes—exemplified by participatory action research and community‐based participatory research—promote stakeholder involvement in decisions affecting their lives (Duckett et al., 2010), aiming for power sharing, mutual learning, and “recogniz[ing] the unique strengths that each brings” (W. F. Kellogg Foundation Community Health Scholars Program, 2001, p. 2). Compared to traditional research, where researchers often monopolize decision‐making and claim expertise, participatory processes amplify the voices of marginalized individuals, strengthen trust in research (Case et al., 2014), and make researchers more self‐reflective and publicly engaged (Israel et al., 1998). In health service development, they can enhance providers' understanding of and service for clients while promoting clients' health literacy and autonomy (Okazaki et al., 2014). Stakeholders' experiential and contextual knowledge also improves research validity and intervention effectiveness, making them more culturally responsive (Collins et al., 2018; Shiu‐Thornton, 2003) and reducing health disparities (Betancourt et al., 2015).

However, in hierarchical contexts, participatory processes can provoke tension or resistance (Dworski‐Riggs & Langhout, 2010). For example, adults resisted children's participation in a U.K. project to design healthy schools (Duckett et al., 2010). To resolve these tensions, Dworski‐Riggs and Langhout (2010) suggest working strategically with existing power structures, increasing community members' “consciousness of power asymmetries,” and “creat[ing] structures that enable community members to challenge those asymmetries” (p. 216). Aside from first‐order changes in solving specific problems, participatory processes may also generate second‐order changes, that is, systemic and structural transformations that “shift power relations to be more egalitarian” (p. 217; see also Perkins et al., 2007). Since studies that analyze participatory processes are limited in number, further research is needed to understand their conditions, challenges, strategies, and outcomes, i.e., first‐ and second‐order changes, in diverse cultural contexts.

In China, participation has been adopted in rural development, urban planning, environment management, and, to a limited extent, public health (e.g., Liu et al., 2011; McKinnon, 2010). For example, Wang et al. (1996) taught rural women in Yunnan to use photo novellas to document and advocate for their health needs. The process enhanced their self‐esteem and disrupted gender‐based exclusion in policymaking. Yet, most Chinese participatory studies emphasize the researcher's perspectives, with few documenting participants' views on the process (except Huang et al., 2024). Moreover, participation has been rarely used in mental health research and service development in China, likely due to the field's prevailing paternalism.

### About this study

What makes participation in mental health service development possible in China? What are the challenges and facilitators for implementing the participatory process? What changes, first‐ or second‐order, may result? This study addresses these questions by analyzing participants' experiences in a case to reveal the complex, context‐specific relationships between variables and perspectives. We examine a project of our own that used participatory processes to develop peer support for persons with SMIs in Guangzhou. This is a unique, not representative, case, as no similar efforts exist in China. Its uniqueness offers in‐depth insight into the conditions that enable participation and the human agency that creates new social realities (Stake, 1995). Beyond China and mental health, our case shows how participatory processes can be adopted in paternalistic societies and contributes to emerging discussions on making such processes accessible and inclusive for persons with disabilities (Huang et al., 2024; McDonald & Stack, 2016; Vaughan et al., 2020).

## METHODS

### Case overview

The project under analysis took place in Guangzhou, an economically prosperous coastal city with the third‐largest population in China. Compared to most other cities, it has a relatively advanced mental health system with diverse services and greater openness to reform. Mainland China's first mental health social work center was founded there in 1999, followed by a dozen similar agencies that provide rehabilitative services to people with SMIs and promote their community inclusion.

In Fall 2018, researchers from a large psychiatric hospital in Guangzhou (hereafter referred to as “the hospital”), which oversees the city's community mental health services, invited the first author, a U.S.‐based academic with prior fieldwork experience in the city, to jointly develop peer support services. In July 2019, they co‐organized a workshop where Chinese and international experts introduced peer support programs. Participants—local psychiatrists, social workers, service users, and family caregivers—were especially interested in a peer navigator program helping to connect homeless African Americans with SMIs to healthcare resources. The program's structured manual and participatory development process stood out (Corrigan et al., 2017), as workshop participants appreciated the concrete guidance provided by the manual and found the participatory process useful for adapting it to the urban Chinese context. Indeed, because workshop participants hoped to have a peer support program to promote social recovery rather than healthcare access for individuals with SMIs, they considered adaptation necessary.

In preparation for the adaptation, local researchers translated the manual, and a bilingual U.S. researcher reviewed and corrected the translation. In December 2019, the first author and two developers of the U.S. program traveled to Guangzhou to lead training on the manual and participatory methods. Trainees included four hospital psychiatrists and researchers, four social workers from two agencies, five service users, and one family member. Service users and the family member were recommended by social workers for their active roles in the communities. Through discussion, participants agreed that the goal of peer support should be to enhance social participation and emotional wellbeing, not health.

After the training, two psychiatrists and three service users withdrew due to scheduling conflicts. Social workers recruited two additional service users, and another social worker joined to prepare for the future implementation of peer support. The team leader, a psychiatrist‐researcher, introduced the participatory approach to the new members. From January to May 2020, the team met every 2 weeks for 2–3 h, typically on Saturday mornings. The meetings initially took place in person at the hospital and were moved online because of the COVID‐19 pandemic. To encourage fuller participation, researchers, social workers, and service users formed sub‐teams to discuss the content in advance of the general meetings; the family member joined the service users' sub‐team. Sub‐team leaders rotated as facilitators of the general meetings, while a hospital psychiatrist handled coordination. Service users and the family member received RMB 40 (about USD 6) per meeting; others were not paid. Throughout, the U.S.‐based researchers provided ongoing consultation. To avoid confusion, only local researchers on the participatory team will be referred to as “researchers” in the results and discussion.

### Data collection

In June 2020, after the manual was adapted, a research assistant unaffiliated with the team interviewed all 12 participatory team members (two psychiatrists‐researchers, five social workers, four service users, and one family member). Interviews took place in private rooms—at the hospital for the psychiatrists‐researchers and at social work agencies for the others. Conducted in Mandarin with verbal consent, each interview lasted about an hour and was audio‐recorded. It covered participants' motivations, perceptions of the team's success, experienced challenges, and suggestions for improved support. No additional compensation was provided. The study received approval from the ethical committees of the participating institutions.

### Data analysis

Interviews were transcribed verbatim in Chinese and checked for accuracy against audio recordings. A research assistant used ATLAS.ti to code the data inductively, identifying themes grounded in participants' lived experiences (Smith et al., 2009). She also compared responses across stakeholder groups to identify commonalities and differences.

The first author met with the RA before coding and weekly thereafter to provide project background and answer questions. She also reviewed a subset of coded transcripts each week, comparing her interpretations with the RA's coding. They discussed discrepancies collaboratively and reached consensus through deliberation. Minor differences in thematic granularity (e.g., combining or splitting subthemes) emerged, but there were no substantive disagreements on thematic content. This iterative process helped ensure reliability and analytic rigor in the final coding structure.

## RESULTS AND DISCUSSION

This section analyzes interview data about the conditions, challenges, facilitators, and outcomes of the participation process. Conditions refer to specific background and contextual factors of the process that may challenge or facilitate participation. Facilitators are strategies that participants identified as helpful during the process or potentially helpful for future efforts. We will also reflect on how these aspects were shaped by the Chinese cultural context, especially professional and familial paternalism, and how they compare with findings from participatory processes in both Western and Chinese contexts.

### Conditions of participation

#### Socio‐demographic characteristics of the participants

As mentioned, the participatory team included two psychiatrists‐researchers, five social workers, four service users, and one family member. Service users ranged in age from 39 to 45 (*M* = 41.25, SD = 2.63), while researchers and social workers ranged from 24 to 44 (*M* = 33.14, SD = 6.72). Three service users (75%) and four professionals (57.14%) were female. The family member was a 69‐year‐old retired woman. None of the service users were employed, and only one had a college degree. In contrast, all professionals and the family member held college degrees or higher.

These disparities reflected the broader service user population in urban China (Wang, 2023). Their socioeconomic disadvantage and lower education levels may have affected their ability to engage with project materials and participate on equal footing with other team members.

#### Team composition

Participants agreed that the stakeholder groups included were relevant to the development and implementation of peer support. Although peer support originated from Western service users' opposition to psychiatry, such antagonism was absent in this project. For example, while one researcher worried that meeting at the hospital might reinforce the doctor‐patient hierarchy and suggested a social work agency instead, others—particularly three social workers and one service user—preferred the hospital due to its convenient location. Family members were also seen as important team members, given their central caregiving role in China and their ability to represent service users' perspectives in ways professionals could not. This contrasts with findings from Canada, where including family members in research sometimes led to conflict with service users (Nelson et al., 1998).

Meanwhile, some professionals on the team recommended involving local government officials. As one researcher put it: “Because service provision has a lot to do with government policy, we need their [government officials’] attention and involvement.” Suggested government sectors included the Disabled Persons' Federation, Civil Affairs Bureau, Political and Legal Affairs Committee, and subdistrict offices. Several participatory health initiatives in China have engaged or collaborated with local authorities, but they noted balancing government support and community leadership as a challenge (Liu et al., 2011; Wang et al., 1996).

Additionally, several participants suggested including diverse service users and family members. One social worker said, “Our team composition met the low bar, but if you want a higher configuration, it should involve different segments of society.” Participants recommended included younger, working, or college‐educated service users, who may face greater stigma and benefit more from peer support, as well as individuals not currently receiving social work services.

#### Reasons and understandings of participation

When asked why they joined the project, most participants mentioned learning about it through the July 2019 conference and trusting the organizers. The hospital was locally reputable, and the first author had established friendly relationships with some participants through prior fieldwork. Professionals were already familiar with peer support through their work. They admired existing programs in China but found them lacking in practice guidelines and service user involvement in program development. By joining the team, they hoped to create a stronger model, advocate for government recognition and funding, and reduce stigma surrounding mental illness. Two professionals were drawn by the participatory approach, while three appreciated the U.S.–China collaboration and the international expertise it brought.

Most service users joined the project with the hope that the peer support program being developed would help improve treatment and rehabilitation services, as well as their access to welfare. Two expressed interest in becoming peer supporters to help others with SMIs and challenge stigma. Three mentioned the modest compensation as appealing. Two others wanted to share their recovery experiences. One explained:

*I experienced big setbacks in my life. But now that I've stood up again, I believe my spiritual power and abilities are stronger than those of people without mental illness. Shouldn't I feel proud about it? I can use this platform to share my successful experience with everyone, including professional psychiatrists and social workers, as well as with peers who are still struggling. This is a pleasurable and meaningful thing*.

Team members saw the project as a way to give voice to service users in designing services, rather than leaving decisions to professionals alone. At the same time, they recognized the importance of professional insight. Some also saw participation as a way to solve community problems and make research more impactful. One social worker emphasized that through program development, service user participants could transform their identities—from recipients to providers of help.

These motivations echo findings from both the U.S. and China: personal relationships and trust were key to engaging community members (Collins et al., 2018; Liu et al., 2011; McKinnon, 2010). In China's paternalistic culture, endorsements from local institutions and international experts lent legitimacy to the project and helped secure buy‐in (Wang et al., 1996).

Moreover, although the biomedical model dominates China's mental health field, participants showed interest in alternatives that counter stigma and marginalization. Serrano‐García (1994) outlined four levels of consciousness regarding power asymmetries: submissive (taking social reality for granted), precritical (dissatisfaction coupled with feelings that a solution is within reach), critical‐integrative (analyzing roots of asymmetries and initiating change efforts), and liberating (seeing asymmetries as oppressive and demanding social transformation). While most people might have a submissive attitude toward existing psychiatric services and the paternalistic culture underneath them, participants in this project demonstrated a precritical consciousness: professionals with a strong sense of responsibility and service users with high self‐efficacy were motivated to break the status quo and pursue peer support as a solution. Together, they were “a committed group of people who come together with the skills and motivation,” an “engine for change” (Evans & Loomis, 2009, 377).

### Challenges of participation

#### Participants' self‐understanding

When asked about their contribution to the team, nearly all participants mentioned providing input on revising the manual based on their experience. However, professionals tended to emphasize their expertise: researchers described guiding the process and gathering information like future funding opportunities, while two social workers noted that their closer contact with service users gave them a more grounded perspective than other professionals.

In contrast, most service users downplayed their roles. Two said they had simply completed tasks assigned by the team leader, and one remarked, “I just gave some suggestions… just the difficulties in everyday life. We all have those, right? I just shared those.” These responses suggest that some service users did not recognize their lived experiences as valuable contributions. As discussed later, such discrepancies in self‐perception may stem from service users' difficulties understanding project materials and reflect their need for greater support in building leadership capacity—especially in light of their long‐standing marginalization.

#### Materials and instructions

Most service users found the manual to be adapted difficult to understand, citing technical language and complex vocabulary. One also expressed discomfort with the phrase “talking about the manual,” preferring clearer and more specific instructions. Similarly, the family member found the discussion‐based training delivered by the U.S. trainers too vague: “In China, if we do this [training], there needs to be a beginning, middle, and end. Their [the trainers'] procedure made you confused about what the order was.”

These critiques echo McMillan's (1975) insights that vague procedures and inadequate guidance from professionals can hinder participation, particularly in inexperienced communities. The hierarchical social system in China likely exacerbated these issues, as nonprofessional participants accustomed to defined roles and responsibilities may struggle with open‐ended discussions and ambiguous activities.

#### Group dynamics

Another challenge raised in interviews was the reticence of service users during meetings. Professionals saw this as a barrier to understanding their needs. The service user sub‐team leader also expressed frustration:

*I would ask them [the other service users] in our small group about how things were going, or whether they had any thoughts about the project. I asked them a lot. I wish they could have been more active so that I didn't have to do all the talking myself*.

Other studies have noted silence and passivity as a challenge. For example, Dworski‐Riggs and Langhout (2010) found that U.S. school staff were passive in participatory team meetings to discuss school change because they were used to receiving, not shaping, information. Sullivan et al. (2005) discovered that despite efforts at inclusivity, participation from the directly affected marginalized populations (e.g., women who experienced domestic violence) remained limited due to emotional unpreparedness from past trauma. In our study, the hierarchical, paternalistic culture likely affected service users' participation. According to some professionals on the team, service users lacked self‐confidence and were afraid of saying anything wrong because they had long been controlled by others. The fact that they were recommended by their providers may have reinforced deference—though, as discussed in Section 3.3, familiarity also enabled provider support.

Professionals and the family member suggested additional reasons for service user reticence: cognitive side effects from medication, lack of clarity about their roles, difficulty understanding the manual, and unfamiliarity with videoconferencing tools. Most of these stemmed from service users' prolonged social marginalization. As one social worker noted, these barriers ultimately prevented service users from participating equally with others, despite the project's inclusive goals.

Note that most service users did not view their reticence as a problem, consistent with their modest understanding of their roles. Moreover, although they spoke less, service users contributed crucial insights that surprised professionals, as discussed in Section 3.4.1.

#### Meeting formats

As mentioned, the team initially met in person before switching to online meetings. Participants identified benefits and drawbacks in both formats. In‐person meetings enabled more direct communication and richer nonverbal cues—such as emotional tone, body language, and eye contact—which professionals valued for supporting service users. As one social worker noted, “Even casual conversations after the meeting are support.” Another said: “When everyone is sitting face‐to‐face, we can easily prompt others to share their thoughts at any time.”

Some professionals found in‐person meetings more formal and meaningful, noting that the commute reinforced the project's importance and could build service users' independent living skills. However, a few participants disliked the long travel time and inefficiency of in‐person meetings. The family member found it hard to raise questions and concerns to the team leader in front of other people, but appreciated the privacy of direct messaging in online meetings. Additionally, a service user felt stuck when meeting face‐to‐face: “Sometimes, when we sat together, I didn't know what to say… It was not stress; it's just that sometimes I couldn't think of anything to say, and many issues didn't come to mind.”

Participants had mixed views on online meetings. Some found them efficient, easier to coordinate, and appreciated how the internet brought people closer during the pandemic. Others found the format distracting, struggled with connectivity issues, or noted that meetings tended to run longer. Views on service users' engagement also varied—some thought they were quieter, while others thought they were more vocal. The service user who struggled in person said: “[Online meetings] are more convenient. Sometimes the questions that don't come to mind face‐to‐face might occur online.”

Overall, service users preferred online meetings for reducing stress and saving travel time. Indeed, existing studies have shown that virtual platforms can help include persons with disabilities by eliminating transportation barriers (Valdez & Gubrium, 2020) and accommodating diverse communication needs (Nicolaidis et al., 2011). The team meetings, as described by participants, resemble what Price (2011) calls “kairotic space,” which pairs spontaneity—real‐time unfolding of events, impromptu communication, in‐person contact, and a strong social element—with (perceived) high stakes. By reducing elements of spontaneity, online meetings can reduce the stress produced by kairotic space.

Meanwhile, some service users lacked adequate equipment or digital literacy, such as a cellphone with sufficient memory or experience with the videoconferencing app, leading to disruptions during online meetings. Thirty years ago, Wang et al. (1996) highlighted the problem of assuming marginalized groups'—in their case, rural women's—access to resources for participation, such as telephones and transportation. The same is still true today, and it reminds us that participation barriers often stem from socioeconomic disadvantage, not individual impairment.

#### Intensity and investments

Participatory processes typically take more time and resources than traditional approaches (Israel et al., 1998). In our case, several participants found the process intense and time‐consuming. The initial 5‐day training—compressed due to the U.S. trainers' tight schedule—left one service user so exhausted she couldn't sleep at night. Afterward, the team met biweekly for 2–3 h. A service user and a researcher found these meetings overwhelming and suggested a 2‐h limit to reduce fatigue, though two social workers preferred longer sessions for deeper discussion. Professionals also struggled to find time to read the manual after work, and weekend meetings cut into their personal time. While most participants agreed the project caused stress and fatigue, they considered it acceptable overall.

Some professionals faced pressure from colleagues who expected immediate results. As one social worker explained:

*They [people in my agency] think participating in this could benefit our work. They expect you to share your insights with everyone if you are involved in this. However, the project hasn't had any results yet, so how can I share anything with you?*

A researcher suggested that greater human resources were needed for coordination and for synthesizing the team's diverse input. She emphasized the importance of institutional support for planning and administration, but the project's limited funding did not cover staff time. When peer support was later piloted in Guangzhou using the adapted manual, these resource constraints made it impossible to continue the participatory process during implementation and evaluation.

### Strategies to facilitate participation

#### Materials and instructions

Initially, the team leader assigned an entire chapter for members to review every 2 weeks. To reduce the workload and improve service user comprehension, she later divided the manual into small sections for daily review and encouraged members to post questions or comments in a social media group (i.e., WeChat). Professionals said this allowed them to read alongside service users and clarify confusing parts. A seasoned social worker said, “I've known them [some service users on the team] for 10 or 20 years, and we've become friends. I would ask them if they could understand a particular sentence. If they couldn't, I would give them some examples.” Service users on the team found such support helpful and hoped for more. One said: “It's easier to understand if there are more real‐life examples; if it's not too vague and more specific.”

Participants recommended additional training to help service users better understand the materials. Suggestions included pre‐screening content for accessibility and offering opportunities to practice abstract concepts. These strategies align with Loignon et al. (2021), who emphasize simplified materials and experiential, multi‐modal learning to support individuals with limited literacy.

#### Group dynamics

To help service users speak up, professionals made a conscious effort to talk less, listen more, show empathy, and offer direct encouragement. A social worker credited this shift to the team leader's guidance:

*At the beginning, we social workers talked too much. It seemed as if we were providing professional feedback, but in fact, that prevented them [service users and the family member] from speaking… I later found that she [the team leader] wanted them to talk more, so I held my tongue*.

A social worker also proposed that, in the future, service users rotate as meeting facilitators—with professionals offering backup—to help reduce their nervousness over time.

Although most service users didn't see their own reticence as a problem, they valued the material, informational, and emotional support from the team. They appreciated having professionals they could consult, as well as their compassion. For example, when one service user couldn't refill medication during a pandemic lockdown, a psychiatrist on the team stepped in to help. Social workers also helped service users from the same agencies navigate the team environment by talking with them on the side about their concerns. One researcher even donated a used computer and cellphone so that service users could join online meetings. Several service users noted these acts with appreciation. One said: “She [the researcher] was so generous that she provided many things [to the project] herself… I think that was great. They [the professionals] were so kind.”

These examples show how professionals participated not only in their official roles but also as supportive individuals and friends. By offering various forms of support, they helped create a more accessible group environment and facilitated participation (Nelson et al., 1998). Service users also supported one another by checking in through WeChat and organizing informal gatherings outside the main meetings. Being able to connect to and support each other during the pandemic not only fostered a sense of solidarity but also enhanced their feelings of belonging and safety during a time of tremendous precarity. Huang et al. (2024) advocate for collective responsibility in ensuring access in participatory research with people with disabilities in China. The mutual support among our participants offers concrete examples of this collective approach.

### Outcomes of participation

#### First‐order change: The manual

The adapted peer support manual—considered the direct output and first‐order change of the participatory process—was approved by all team members. They found it comprehensive and especially appreciated its coverage of practical skills, such as responding to clients' concerns, engaging individuals, and teaching everyday living skills. It was seen as a clear improvement over the original version because it incorporated local stakeholders' suggestions, particularly service users' lived experiences and needs. For instance, service users' confusion with the original manual helped the team identify areas for revision, improving clarity and accessibility. Their feedback also challenged professionals' assumptions about their capabilities and priorities. As one researcher explained:

*We [professionals] thought that they [service users] needed knowledge to become peer supporters, but they had a lot of practical questions. For example, we considered home visits to be a small element of peer support, but they were concerned about the safety of peer supporters conducting the visits. They provided numerous suggestions, including guidelines on how to pair up and how to evaluate the situation. They were incredible*.

Another researcher praised the manual's contextual relevance: unlike the original version, the adapted manual de‐emphasized navigating health resources—since service users in urban areas typically had access to doctors—and added guidance on managing client aggression, a widespread concern. By integrating locally situated experiences and centering marginalized voices, the participatory process enhanced the manual's cultural responsiveness (Shiu‐Thornton, 2003).

Nevertheless, professionals and the family member identified problems in the adapted manual. Some social workers found the manual overly detailed and demanding for peer supporters, noting that even professionals may struggle to connect service users to various resources. One commented: “If we use this [high] standard [set by the manual], social workers need to spend much time training them [peer supporters], and it might still not be effective.” In addition, a social worker and the family member found parts of the adapted manual too conceptual: whereas the original version offered concrete steps on connecting clients to health resources, such as making appointments, the adapted version contained more abstract discussions of resources. Service users did not identify specific problems with the manual during the interview, but they recommended adding more content on practical skills, such as addressing medication side effects and maintaining client relationships.

These issues likely stemmed from the greater influence professionals had in the adaptation process, despite efforts to share power (Israel et al., 1998), and their ease with technical content. The team's ambition for comprehensive peer services and the lack of clarity about how the manual would ultimately be used—since peer support had yet to be implemented—also contributed to the problems. These limitations underscore the need for iterative service development and continued use of participatory approaches throughout implementation, evaluation, and revision.

#### Toward second‐order changes: Consciousness, capacity, and relationships

As Dworski‐Riggs and Langhout (2010) argue, in communities where shared decision‐making is uncommon, participatory processes may serve as interventions that challenge boundaries and “create conditions that foster empowerment and initiate second order change” (p. 226). While our project did not result in permanent institutional restructuring or a redistribution of service control, interview data reveal several intermediate and process‐level outcomes that community psychologists consider important precursors to such change (Brown, 2009; Evans & Loomis, 2009; Fang et al., 2024; Perkins et al., 2007).

First, participants gained new knowledge and capabilities. According to the interviews, professionals learned how to construct participatory processes and reflected on how to integrate them into their work. They acquired skills in active listening and shifted their orientation from directing to supporting service users. Service users gained knowledge about peer support, improved communication skills, and began to view their experiences as valuable expertise. These developed capacities reflect early stages of empowerment, which can lay the groundwork for more structural changes in roles and responsibilities (Brown, 2009).

Second, participants expressed increased power awareness and reflexivity. One researcher reflected, “It is not the relationship of subordination common between doctors and patients in China. It is very equal.” A social worker stated, “I now know that service users are the protagonists and we social workers are in supportive roles.” These reflections signal a shift from precritical to critical‐integrative or liberatory consciousness—awareness of oppressions and efforts to make changes (Serrano‐García, 1994). While insufficient on their own, these shifts are necessary building blocks for systemic transformation (Evans & Loomis, 2009).

Third, service users reported increased self‐worth, purpose, and social connectedness. One service user appreciated the intellectual stimulation of the project, while social workers observed that the team's clear goals offered service users a renewed sense of purpose. Another service user felt less alone upon discovering others faced similar life challenges. A third expressed gratitude for the professionals' attention and respect:

*I feel that… professionals are leaning more and more toward us who have mental illnesses. They give us a lot of positive energy and resources. In return, I should live in a more meaningful and fulfilled way, to share my experience with everyone*.

These findings show that, while professionals began to learn from and support service users, service users also began to feel needed by professionals and became more confident in contributing their wisdom to service development. This shift marked a transformation in identity: from passive recipients of care to active contributors and collaborators in shaping services. Notably, the contributions of different parties remained uneven: professionals had a greater ability to share resources, and their expertise was still generally held in higher regard. This imbalance highlights how hierarchy and structural inequality continued to shape the process. Nevertheless, participation began to foster mutual, not one‐way, support and generate relationships of interdependency and solidarity (Huang et al., 2024; Nelson et al., 1998). Such relational and identity transformations have been shown to support long‐term engagement in leadership and advocacy (Davidson et al., 2012; Fang et al., 2024), especially when paired with organizational and social support for sustained involvement from a broader range of stakeholders in participatory processes.

## CONCLUSIONS

This study represents one of the first systematic attempts to implement and analyze a participatory process in mental health service development in China. By engaging diverse stakeholders—especially people with serious mental illnesses—as collaborators rather than subjects, we demonstrated that inclusive, trust‐based participation is both possible and productive even within a paternalistic system. The process not only generated a culturally responsive service model but also new forms of reflection, recognition, and relationship among service users and professionals. Meanwhile, sustained institutional commitment to participation and expanded structural opportunities for service users to exercise leadership are needed for broader transformations.

Drawing on participant perspectives, this study examined the conditions, challenges, facilitators, and outcomes of participatory processes in China's mental health field. Personal relationships and trust, as well as endorsements from local institutions and international experts, were crucial for engaging community members and legitimizing this new approach. Despite a paternalistic mental health system, participation was made possible by a group of people dissatisfied with the status quo, who had the sense of responsibility and self‐efficacy to pursue change. The relatively diverse and reform‐minded mental health landscape in Guangzhou may have also encouraged openness. Moreover, the participatory process involved service users, family members, social workers, and psychiatrists/public health researchers—stakeholders central to the development of peer support. Meanwhile, given the government's significant influence on social services, future efforts would benefit from involving government officials. More diverse community representatives were also desired, although the scarcity of changemakers—at least at the initial stage—and the reliance on personal networks for recruitment made this difficult.

Service users faced challenges with theoretical or technical materials due to their low educational levels. They also felt overwhelmed by long meetings, large tasks, and ambiguous activities. These difficulties and their long‐standing marginalization contributed to their limited engagement and modest self‐understanding in participation. Online meetings helped reduce stress and travel time while accommodating diverse communication styles, but technological barriers and professionals' difficulty in responding to service users' emotional needs also emerged. In addition, professionals faced institutional disincentives such as a lack of recognition or support for participatory work.

To enhance participation, especially that of service users, it was useful to space meetings adequately, assign manageable tasks, and provide clear instructions. Professionals facilitated engagement by reviewing materials with service users, simplifying language, actively listening to their thoughts and concerns, and offering direct encouragement. Social workers helped service users navigate the team environment, and professionals also addressed their health and material needs. Service users, in turn, offered emotional support to one another through WeChat groups and informal gatherings. These strategies fostered inclusion but were insufficient to ensure equal participation. Future efforts should include additional training for service users, pre‐screening materials for accessibility, and structured opportunities for them to practice leadership roles.

The participatory process yielded a culturally responsive service manual tailored to the urban Chinese context and the needs of stakeholders. Compared to the original version, it emphasized practical skills—such as managing client aggression—while reducing focus on navigating healthcare resources that were already accessible locally. Yet the manual remained somewhat conceptual and ambitious, partly because professionals exerted greater influence, and institutional support was limited for iterative manual improvement during service implementation. Besides this tangible outcome, the participatory process also fostered shifts in consciousness, identity, and relationships. Professionals developed a stronger awareness of service users' lived experiences and structural marginalization, adopting more supportive, less directive roles. Service users gained a sense of purpose, fulfillment, and recognition, beginning to see themselves as contributors and collaborators. Although power imbalances persisted, these evolving understandings and relational practices represent crucial precursors to second‐order change.

This case offers insight into implementing participatory processes in paternalistic contexts and with disabled populations. First, it shows the utility of leveraging personal relationships and institutional endorsements to initiate participation, while recognizing that these may limit inclusivity and reinforce power imbalance. Second, in paternalistic contexts, involving family members can strengthen participation if their voices complement rather than substitute those of service users. Similarly, hierarchies between professionals (or other power holders) and marginalized community members need not produce antagonism; instead, participatory processes can foster mutual recognition, learning, and support, cultivating professionals' critical reflexivity and empowering community members. Third, recognizing and addressing the complex needs rooted in psychiatric service users' marginalization is essential for enhancing participation. While our team implemented several strategies to accommodate these needs, they were only partially effective. Persistent hierarchies, limited service user influence, and insufficient organizational support reveal the limitations of small‐scale participatory efforts in addressing systemic inequities. While valuable for individual growth and collective understanding, lasting transformation requires sustained institutional commitment and structural reforms.

Our case study has several limitations. First, compared to professionals, service users spoke less during interviews, perhaps hesitant to criticize the process. Further efforts are needed to empower them and emphasize the value of their perspectives. Second, as a unique case, the study illustrates what is possible for participatory service development in a paternalistic context, but it may not represent broader stakeholder views. Future research should assess general attitudes toward participation, especially among underrepresented groups such as male professionals and family members, as well as younger, employed, or more educated service users. Third, our interviews were conducted immediately after the project was completed. Longitudinal studies are needed to assess whether changes in consciousness, capacity, and relationships endure—and whether they can facilitate broader transformation.
